# Supplementary material for: SLMO transfers phosphatidylserine between the outer and inner mitochondrial membrane in Drosophila
Source: PLoS Biol. 2024 Dec 16;22(12):e3002941. doi: 10.1371/journal.pbio.3002941 (PMC11649117; doi:10.1371/journal.pbio.3002941)
Supplement: S1 Table — (PDF) [file pbio.3002941.s001.pdf]

S1 Table: Screen results

#SUBMITTED ID	NAME
CG7889	-
CG7834	Electron transfer flavoprotein beta subunit
CG3499	YME1 like ATPase
CG33052	Golgin, RAB6 interacting
CG4501	bubblegum
	Inositol 1,4,5-triphosphate kinase 2
CG9977	Adenosylhomocysteinase like 1
CG8553	Selenophosphate synthetase 1
CG6050	mitochondrial translation elongation factor Tu 1
CG9484	hyperplastic discs
CG6852	Glutaredoxin 1
CG16916	Regulatory particle triple-A ATPase 3
CG10121	SP1173
CG31150	crossveinless d
CG17486	-
CG5322	Lysosomal alpha-mannosidase I
CG6664	-
CG4501	bubblegum
	Inositol 1,4,5-triphosphate kinase 2
CG9977	Adenosylhomocysteinase like 1
CG11154	ATP synthase, beta subunit
CG9131	slowmo
CG2095	Secretory 8
CG2023	Secretory 20
CG4572	-
CG4157	Regulatory particle non-ATPase 12
CG1349	dj-1beta
CG31414	Glucocerebrosidase 1b
CG32434	schizo
CG1906	alphabet
CG9796	Gamma-interferon-inducible lysosomal thiol reductase 1
CG8426	CCR4-NOT transcription complex subunit 3
CG32113	Vacuolar protein sorting 13D
CG2446	Amun
CG2520	like-AP180
CG10778	Dehydrodolichyl diphosphate synthase subunit
CG7757	Precursor RNA processing 3
CG1871	enhancer of rudimentary
CG10693	slowpoke
CG4945	-
CG10158	Fibroblast growth factor receptor 1 oncogene partner 2
CG33115	Nimrod B4

CG11334	-
CG4916	maternal expression at 31B
CG5322	Lysosomal alpha-mannosidase I
CG4422	GDP dissociation inhibitor
CG11154	ATP synthase, beta subunit
CG2023	Secretory 20
CG1349	dj-1beta
CG31414	Glucocerebrosidase 1b
CG1906	alphabet
CG9796	Gamma-interferon-inducible lysosomal thiol reductase 1
CG8654	-
CG1685	penguin
CG14028	cyclope
CG16705	Spatzle-Processing Enzyme
CG8417	Mannose phosphate isomerase
