# Supplementary material for: SLMO transfers phosphatidylserine between the outer and inner mitochondrial membrane in Drosophila
Source: PLoS Biol. 2024 Dec 16;22(12):e3002941. doi: 10.1371/journal.pbio.3002941 (PMC11649117; doi:10.1371/journal.pbio.3002941)
Supplement: S2 Table — (PDF) [file pbio.3002941.s002.pdf]

S2 Table: qPCR validation of indicated RNAi lines.

qPCR assay	GMR-GAL4	GMR> UAS-RNAi	p-value
slmo RNAi1	1	0.146179649	0.0007
slmo RNAi2	1	0.43533861	0.0037
slmo RNAi	1	0.644625212	0.0381
slmo RNAi5	1	0.535404805	0.0075

UAS-RNAi lines were crossed with GMR-gal4, and adult eyes of F1 flies were dissected for RNA extraction and subsequent qPCR. Samples were prepared in triplicate. All values were normalized to GMR-gal4 samples. *Rp49* was used as the reference gene for standardization. The p-values were obtained using a Student's T-tests against the GMR-gal4 samples.

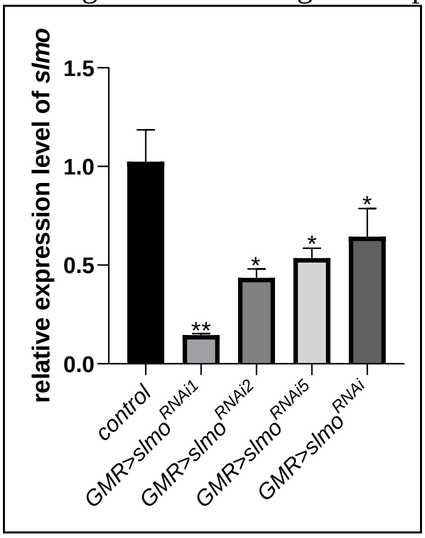

RNA interference in S2 cells

qPCR assay	*BFP RNAi*	*X RNAi*	p-value
slmo	1	0.309445	0.01517626
pisd	1	0.64266879	0.27258558
pss	1	0.76371597	0.10701278

S2 cells were transfected with long dsRNAs against *BFP*, *slmo*, *pisd* and *pss* for 3 days, and subsequent qPCR. All values were normalized to *BFP*. *Rp49* was used as the reference gene for standardization.

RNA interference in Hela cells

qPCR assay	*control*	*Slmo2 RNAi*	p-value
Slmo2 RNAi1	1	0.14481773	0.01588503
Slmo2 RNAi2	1	0.6950063	0.0515901

HELA cells were transfected with lentiviral shRNAs to generate stable RNAi lines, and subsequent qPCR. All values were normalized to shcon. GAPDH was used as the reference gene for standardization.
