# Supplementary figures and images for: SLMO transfers phosphatidylserine between the outer and inner mitochondrial membrane in Drosophila
Source: PLoS Biol. 2024 Dec 16;22(12):e3002941. doi: 10.1371/journal.pbio.3002941 (PMC11649117; doi:10.1371/journal.pbio.3002941)

Fig 4B

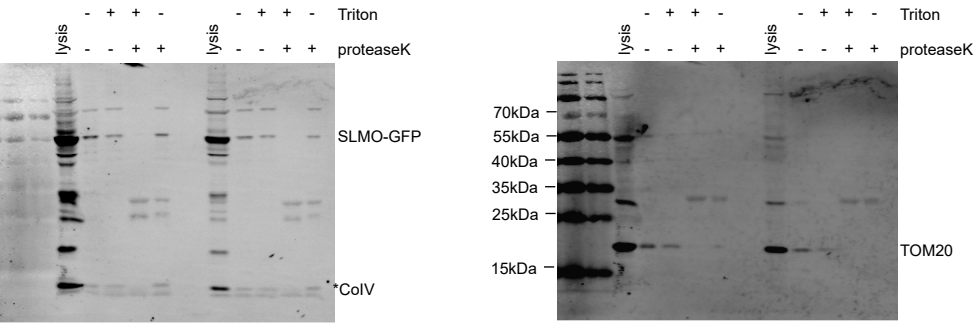

Fig 6I

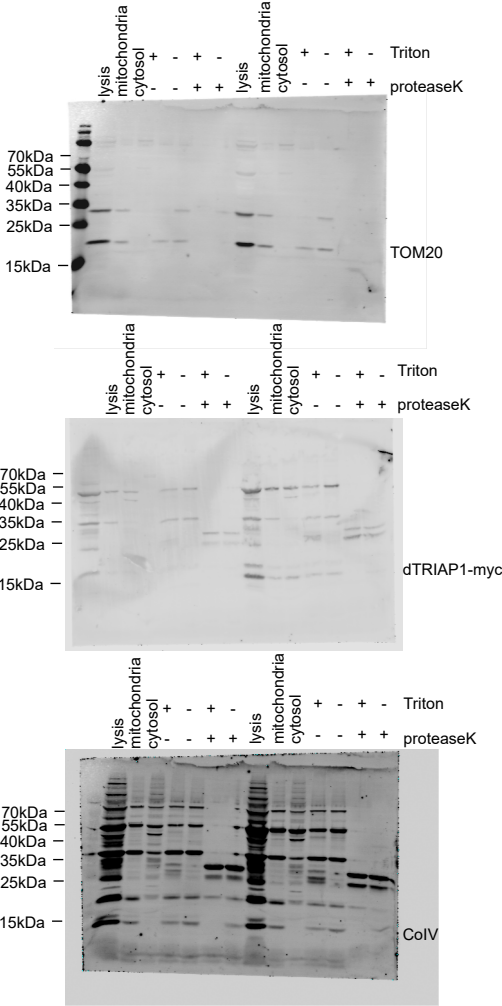

Fig S4D

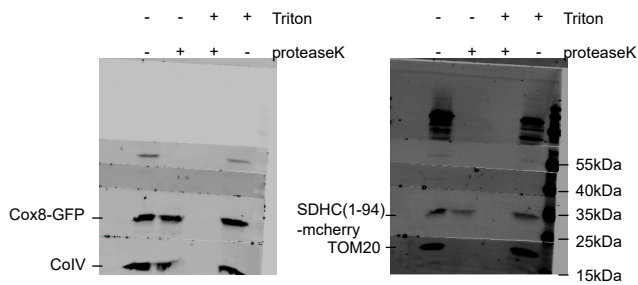

Fig S5A

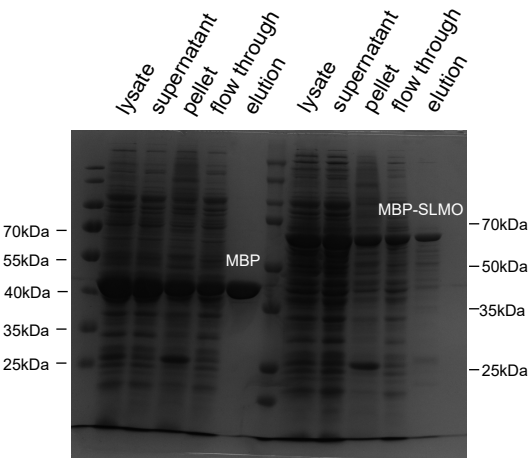

Fig S5B

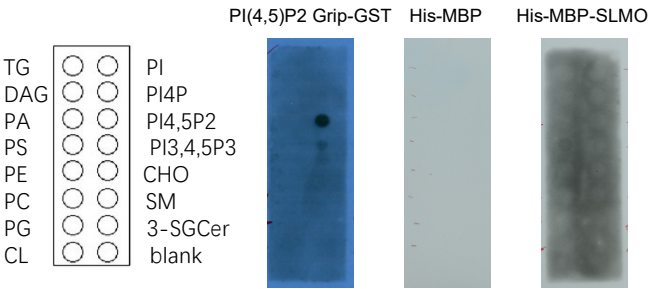

Fig S5C

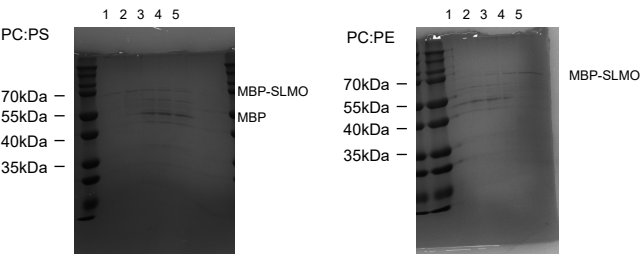

Fig S5E

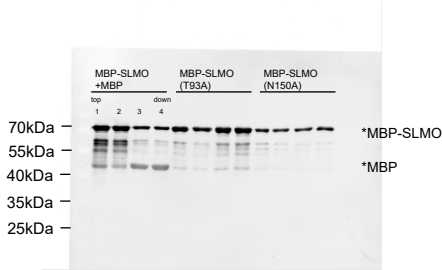

Fig S5G

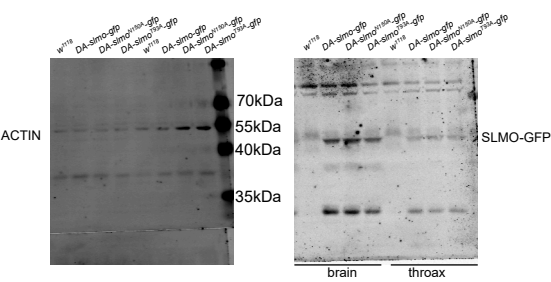

RAW data

Fig S7E

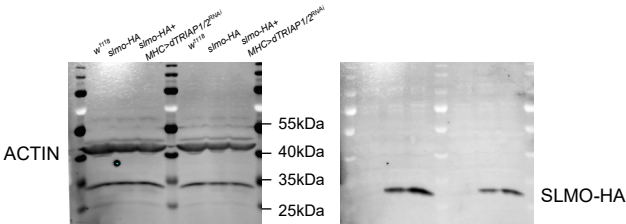

Supplement: S1 Raw Images — (PDF) [file pbio.3002941.s004.pdf]

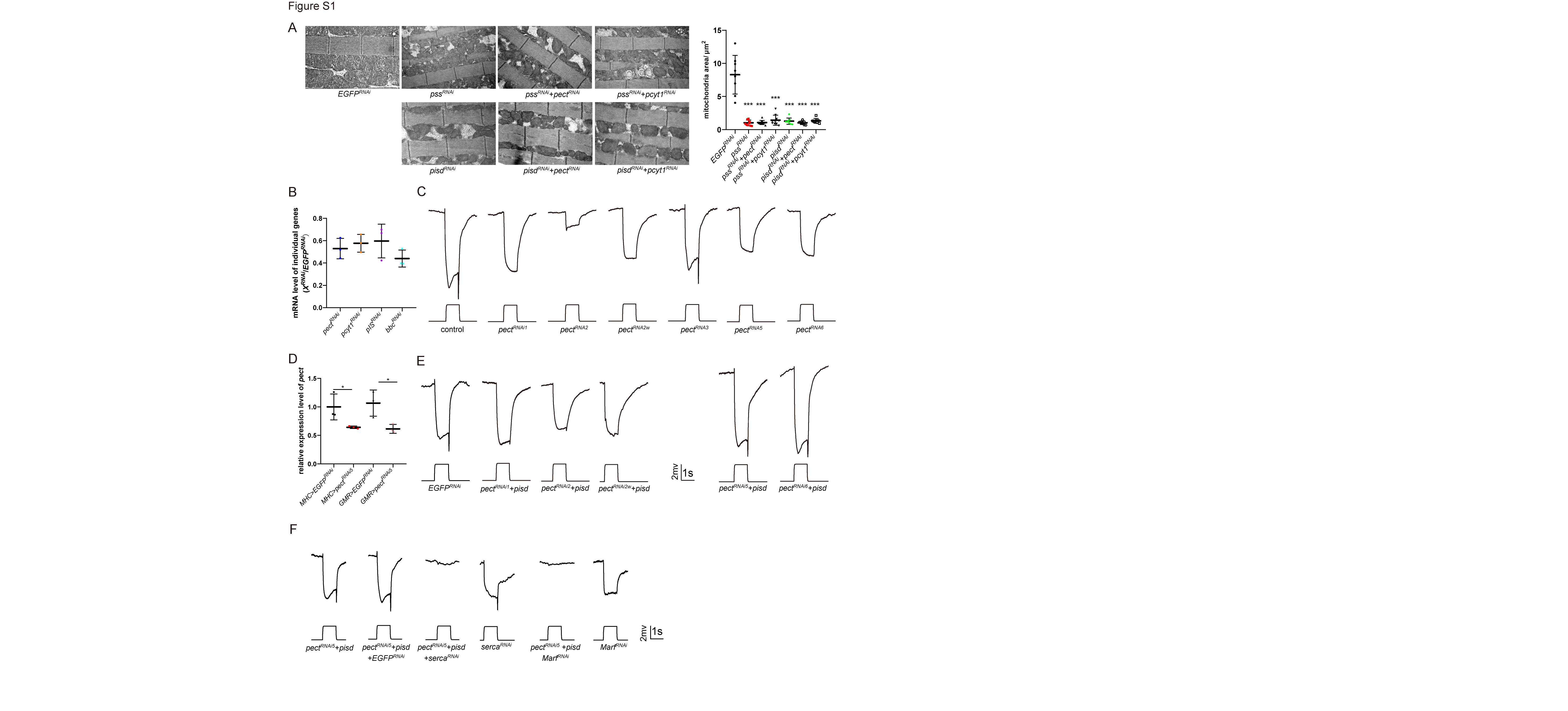

Supplement: S1 Fig — (A) pect or pcyt1 deficiency failed to rescue pssRNAi and pisdRNAi. Muscle sections from EGFPRNAi (MHC-gal4/UAS-EGFPRNAi), pssRNAi (MHC-gal4/+;UAS-pssRNAi/+), pssRNAi+pectRNAi (MHC-gal4/+;UAS-pssRNAi/UAS-pectRNAi), pssRNAi+pcyt1RNAi (MHC-gal4/+;UAS-pssRNAi/UAS-pcyt1RNAi), pisdRNAi (MHC-gal4/+;UAS-pisdRNAi/+), pisdRNAi+pectRNAi (MHC-gal4/+;UAS-pisdRNAi/UAS-pectRNAi), and pisdRNAi+pcyt1RNAi (MHC-gal4/+;UAS-pisdRNAi/UAS-pcyt1RNAi) flies. Scale bar, 1 μm. Mitochondria size of at least 6 samples of each phenotype were quantified. (B) The ratio of mRNA level of pect, pcyt1, pis and bbc in MHC>pectRNAi, MHC>pcyt1RNAi, MHC>pisRNAi, and MHC>bbcRNAi individually to MHC>EGFPRNAi. Total RNA was extracted from the dissected fly thorax of MHC>EGFPRNAi, MHC>pectRNAi, MHC>pcyt1RNAi, MHC>pisRNAi, and MHC>bbcRNAi. The relative expression of target genes was normalized to RP49, which serves as an internal control. Data are presented as mean ± SD, *p < 0.05 (Student’s unpaired t test). n = 3. (C) ERG recordings from 5-day-old control (EGFPRNAi, GMR-gal4/UAS-EGFPRNAi), pectRNAi1 (GMR-gal4/UAS-pectRNAi1), pectRNAi2 (GMR-gal4/UAS-pectRNAi2), GMR>pectRNAi2w (GMR-gal4/UAS-pectRNAi2w), GMR>pectRNAi3 (GMR-gal4/UAS-pectRNAi3), GMR>pectRNAi5 (GMR-gal4/UAS-pectRNAi5), and GMR>pectRNAi6 (GMR-gal4/UAS-pectRNAi6) flies. Flies were exposed to a 5-s pulse of orange light after 2 min of dark adaptation. (D) mRNA level of pect was calculated of pectRNAi in muscle and retina. Total RNA was extracted from the dissected fly thorax and retina of MHC>EGFPRNAi, MHC>pectRNAi5, GMR>EGFPRNAi, and GMR>pectRNAi5. The relative expression of target genes was normalized to RP49, which serves as an internal control. Data are presented as mean ± SD, *p < 0.05 (Student’s unpaired t test). n = 3. (E) Overexpression of PISD rescued the defects caused by pectRNAi in ERG response. Five-day-old EGFPRNAi (control), pectRNAi1+pisd (trp-pisd GMR-gal4/UAS-pectRNAi1), pectRNAi2+pisd (trp-pisd GMR-gal4/UAS-pectRNAi2), pectRNAi2w+ [file pbio.3002941.s005.tif]

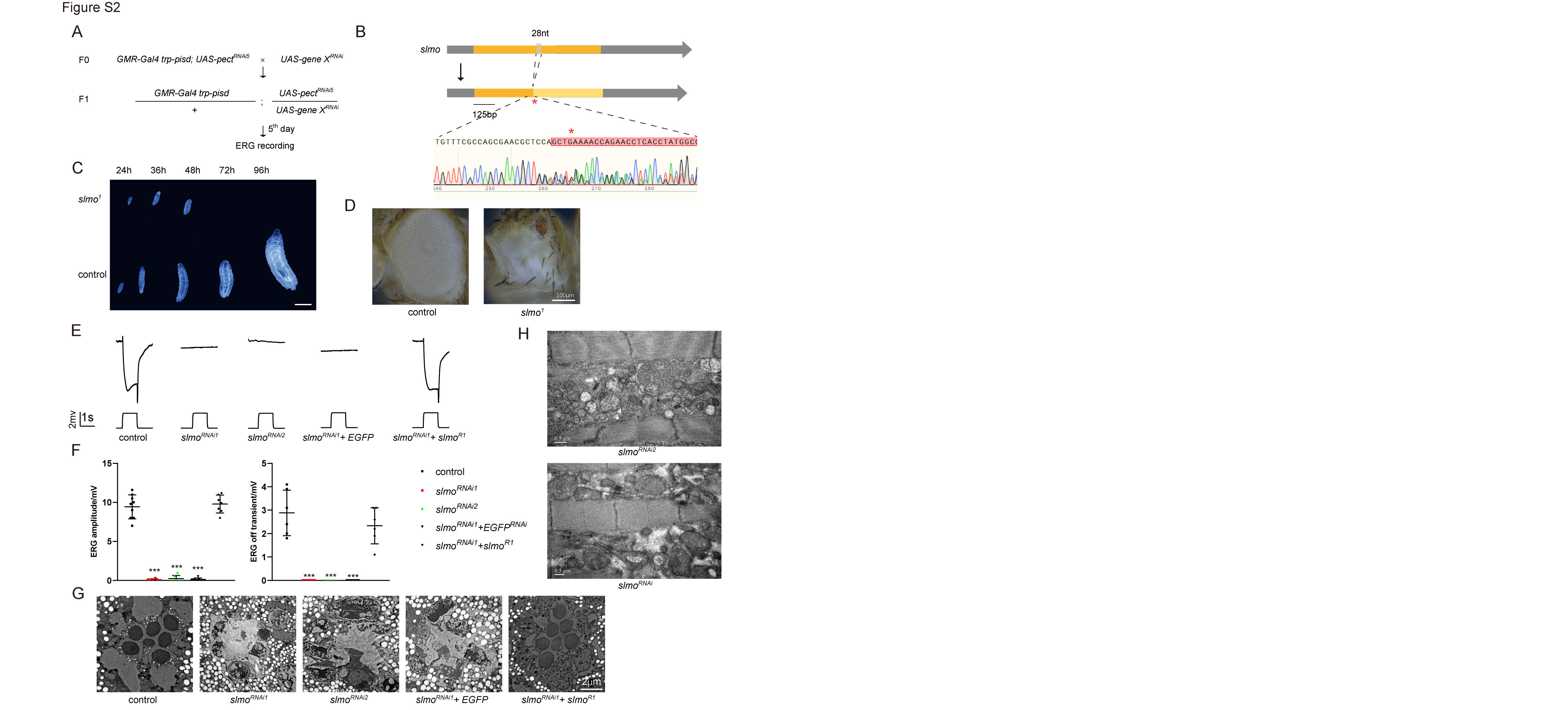

Supplement: S2 Fig — (A) Schematic of the genome-wide RNAi screen for factors that prevent the rescue effects of PISD on the ERG defects of pectRNAi. (B) The slmo locus and mutation site are associated with slmo1. DNA sequencing revealed that slmo1 eliminates 28 bp within the slmo coding region. (C) slmo1 mutants are growth arrested. Images of slmo1 homozygous mutant larvae at different stages (24–96 h) of larval development. w1118 was used as a wild-type control. The scale bar is 100 μm. (D) Light microscope images of eyes of 1-day-old w1118 and slmo1 (ey-flp;slmo1 FRT40A/GMR-hid CL FRT40A) flies. The scale bar is 100 μm. (E) Strong slmoRNAi lines induced severe functional and structural defects in photoreceptor cells. ERG recordings from 1-day-old control (GMR-gal4/UAS-EGFPRNAi), slmoRNAi1 (GMR-gal4/UAS-slmoRNAi1), slmoRNAi2 (GMR-gal4/UAS-slmoRNAi2), and slmoRNAi1+slmoR1 (GMR-gal4/UAS-slmoRNAi1 UAS-slmoR1/+) flies. Flies were exposed to a 5-s pulse of orange light after 2 min of dark adaptation. (F) The amplitudes and off-transients of ERG responses were quantified. At least 10 flies were used, and significant differences were determined using the unpaired t test. (G) TEM retinal sections were obtained from 5-day-old flies of the same genotype as (E). The scale bar is 2 μm. (H) TEM sections of muscles from slmoRNAi2 (MHC-gal4/UAS-slmoRNAi2) and slmoRNAi (MHC-gal4/UAS-slmoRNAi) flies show decreased mitochondrial size and damaged cristae. The scale bar is 0.5 μm. The data underlying the graphs shown in the figure can be found in S2 Table. (TIF) [file pbio.3002941.s006.tif]

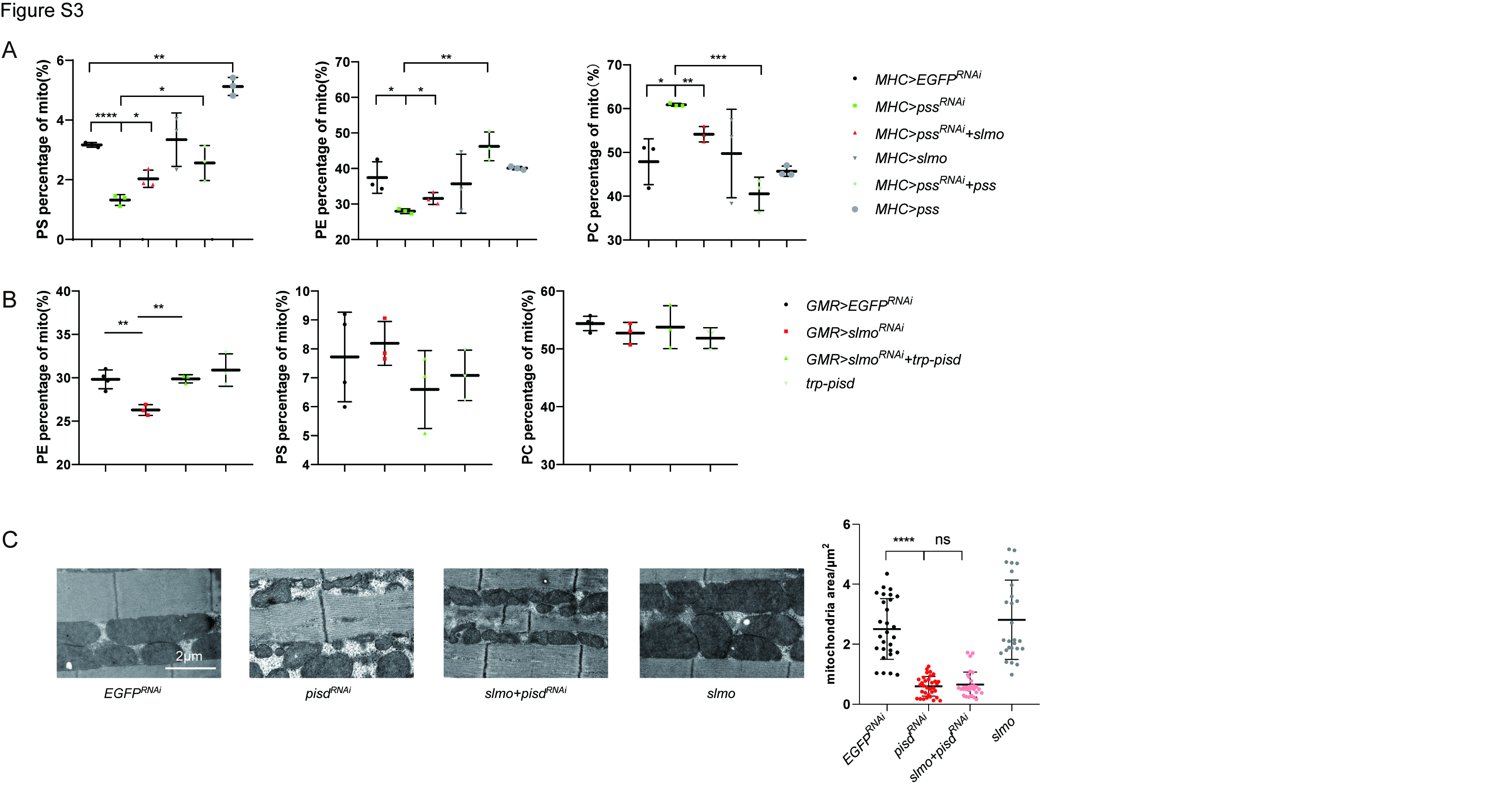

Supplement: S3 Fig — (A) Lipidomic analysis of mitochondrial PC, PE, and PS levels of control (EGFPRNAi, MHC-gal4/UAS-GFPRNAi), pssRNAi (MHC-gal4/UAS-pssRNAi), pssRNAi+slmo (MHC-gal4/UAS-pssRNA;UAS-slmo), slmo(MHC>UAS-slmo), pssRNAi+pss (MHC-gal4/UAS-pssRNAi;UAS-PSS), and pss (MHC-gal4/UAS-pss) muscles. Mitochondria were isolated from 10 dissected thoraxes per assay, and 3 replicates were quantified. (B) Overexpression of pisd restored mitochondrial PE levels the slmoRNAi retina. Lipidomic analysis of mitochondrial PC, PE, and PS levels of EGFPRNAi (GMR-gal4/UAS-GFPRNAi), slmoRNAi (GMR-gal4/UAS-slmoRNAi), slmoRNAi+trp-pisd (GMR-gal4/UAS-slmoRNA;trp-pisd), and trp-pisd photoreceptor cells. Mitochondria were isolated from 60 dissected thoraxes per assay, and 3 replicates were quantified. (C) SLMO overexpression failed to reverse decreased mitochondrial size from pisdRNAi lines. TEM sections of muscles from EGFPRNAi, pisdRNAi (MHC-gal4/UAS-pisdRNAi), slmo (MHC-gal4/UAS-slmo), and slmo+pisdRNAi (MHC-gal4/UAS-slmo;UAS-pisdRNAi) flies. The scale bar is 2 μm. Mitochondria size was quantified and significant differences were determined using the unpaired t test. (TIF) [file pbio.3002941.s007.tif]

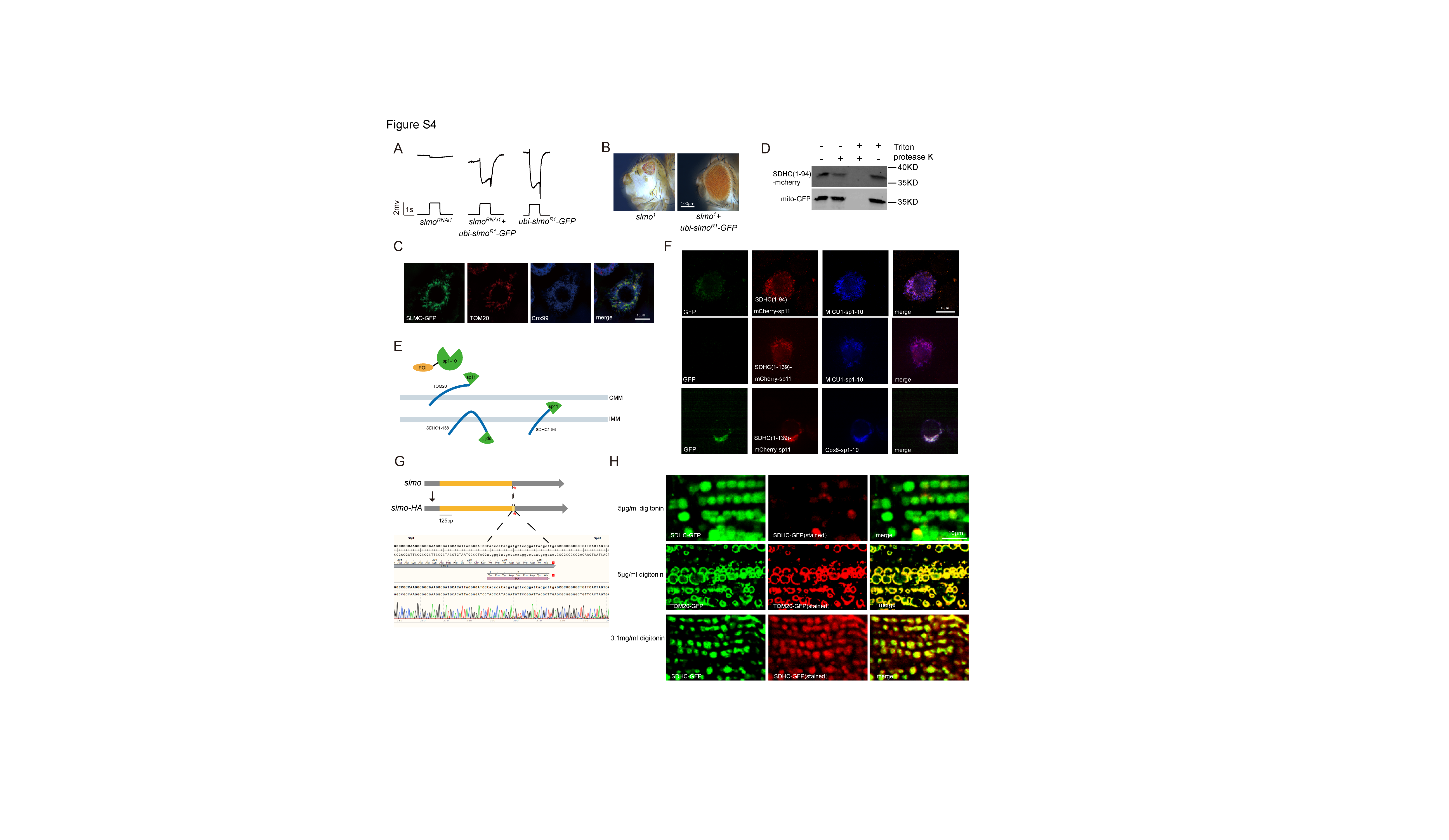

Supplement: S4 Fig — (A) ERG recordings from 5-day-old slmoRNAi1 (GMR-gal4/UAS-slmoRNAi1), slmoRNAi1+ubi-slmoR1-GFP (GMR-gal4/+;UAS-slmoRNAi1/ubi-slmoR1-GFP), and ubi-slmoR1-GFP (ubi-slmoR1-GFP) flies. (B) Light microscope images of eyes of 1-day-old slmo1 (ey-flp;slmo1 FRT40A/GMR-hid CL FRT40A) and slmo1+ ubi-slmoR1 (ey-flp;slmo1 FRT40A/GMR-hid CL FRT40A;ubi-slmoR1-GFP/+) flies. The scale bar is 100 μm. (C) S2 cells expressing SLMO-GFP were imaged using confocal fluorescent microscopy. TOM20 (red) and CNX99 (blue) were stained to visualize mitochondria and ER, respectively. Scale bar, 10 μm. (D) Cell lysates from S2 cells transfected with SDHC(1–94)-mCherry and mito-GFP (Cox8-Sod2-GFP, N-terminal sequences form Cox8 and SOD2 drives GFP to mitochondrial matrix) were analyzed using the protease K protection assay. Without Triton X-100, both mCherry and GFP were resistant to protease K treatment. (E) Design of the split-GFP system. spGFP1-10 was fused to the protein of interest (POI) and spGFP11 was fused to TOM20, SDHC(1–94), or SDHC(1–139). When the POI and spGFP11 were on the same side of the membrane, spGFP1-10 and spGFP11 bind and emit green fluorescence. (F) Verification of split-GFP system in S2 cells. S2 cells co-expressing sp1-10-tagged IMS protein MICU1 (MICU1-sp1-10) and matrix protein Cox8 with SDHC(1–94)-mCherry-sp11 or SDHC(1–139)-mCherry-sp11, were directly imaged for mCherry (red) and GFP (green) fluorescence using confocal fluorescent microscopy. The sp1-10 tag was stained with rabbit-GFP antibody (blue) as well. Scale bar, 10 μm. (G) Schematic diagram of the slmo-HA knock-in strategy. slmo-HA knock-in flies were verified via genomic DNA sequencing. (H) Muscle tissues from ubi-SDHC-GFP and ubi-TOM20-GFP were penetrated by 5 μg/ml or 0.1 mg/ml digitonin, and the samples were stained for TOM20-GFP/SDHC-GFP (red), and GFP signals were directly observed (green). Scale bar, 10 μm. (TIF) [file pbio.3002941.s008.tif]

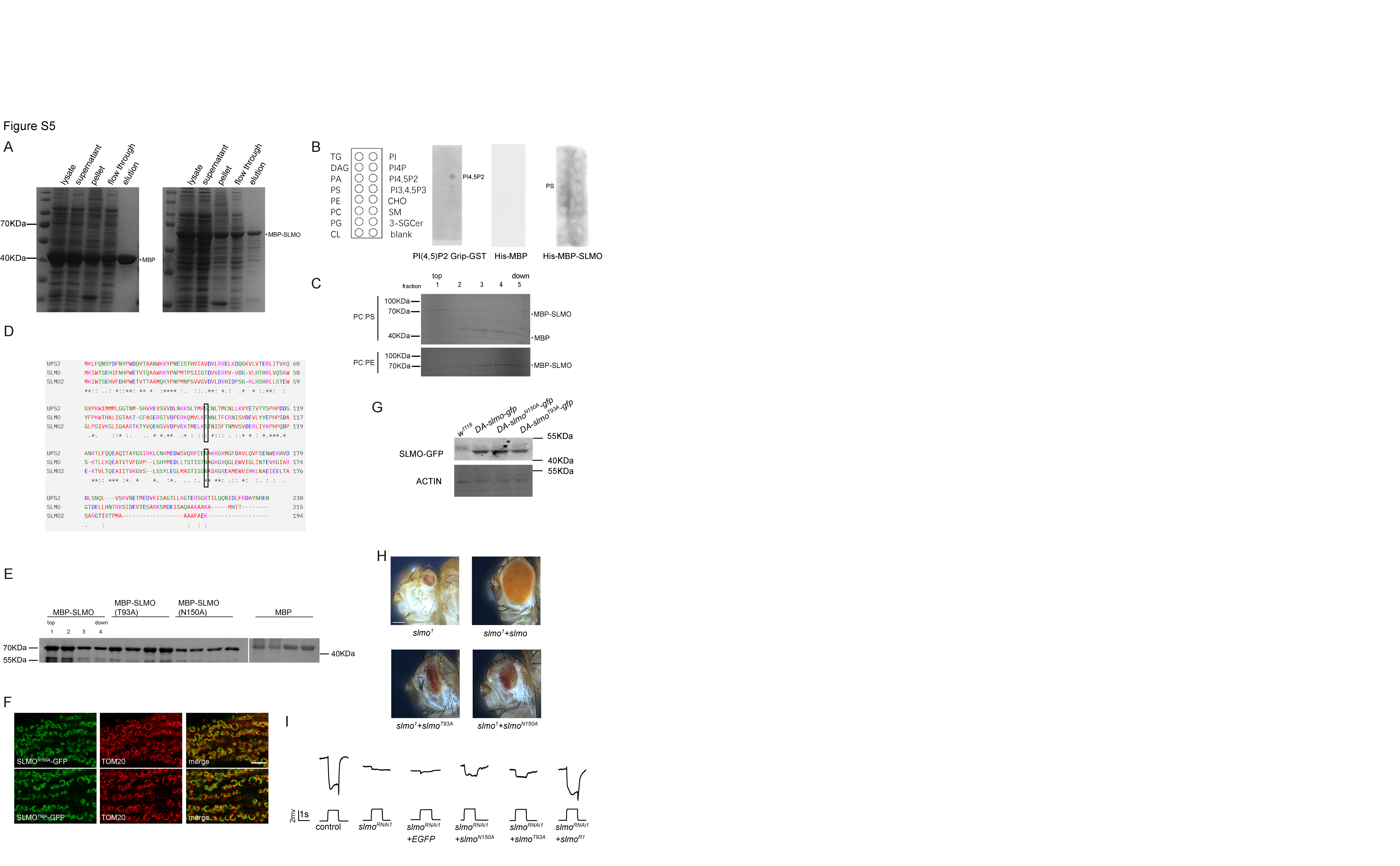

Supplement: S5 Fig — (A) Purification of MBP-SLMO and MBP from E. coli cell lysates. The supernatant, pellet, flow through, and elution were analyzed by SDS–PAGE. Asterisks (*) mark MBP-SLMO and MBP. (B) A schematic diagram of the lipid membrane strip containing the indicated phospholipids is shown on the left. Grip-GST, which specifically binds PI (4, 5) P, was used as a positive control. MBP-SLMO specifically interacts with PS, and MBP alone did not interact with any phospholipid. (C) Liposome flotation assay shows that SLMO binds PS. The supernatants and pellets (100 μl of each sample, from top to down) were subjected to Tricine-SDS PAGE analysis. (D) Amino acid alignment of yeast UPS2, SLMO (Drosophila), and SLMO2 (Human) using the ClustalW Multiple Sequence Alignment program. Identical and conserved residues are indicated with asterisks and dots, respectively. T93A and N150A are circled. (E) Liposome flotation assay of SLMO and 2 mutants. T93A and N150A both reduced the binding affinity between SLMO and PS. (F) Muscle tissues from DA-slmoN150A-GFP or DA-slmoT93A-GFP were dissected and stained with TOM20 (red) and GFP (green). Scale bar, 10 μm. (G) Western blot analysis of proteins extracted from the heads of w1118, DA-slmo-GFP, DA-slmoN150A-GFP, and DA-slmoT93A-GFP flies with antibodies against GFP. Actin was used as a loading control. (H) Light microscope images of eyes of 1-day-old slmo1 (ey-flp;slmo1 FRT40A/GMR-hid CL FRT40A), slmo1+slmoN150A (ey-flp;slmo1 FRT40A/GMR-hid CL FRT40A;DA-slmoN150A/+), slmo1+slmoT93A (ey-flp;slmo1 FRT40A/GMR-hid CL FRT40A;DA-slmoT93A/+), and slmo1+slmo (ey-flp;slmo1 FRT40A/GMR-hid CL FRT40A;DA-slmo/+) flies. Scale bar, 100 μm. (I) ERG recordings from 5-day-old control (GMR-gal4/EGFPRNAi), slmoRNAi1 (GMR-gal4/UAS-slmoRNAi1), slmoRNAi1+EGFP (GMR-gal4/+;UAS-slmoRNAi1/UAS-EGFP), slmoRNAi1+slmoN150A (GMR-gal4/+;UAS-slmoRNAi1/DA-slmoN150A), slmoRNAi1+slmoT93A (GMR-gal4/+;UAS-slmoRNAi1/DA-slmoT93A) flies. (TIF) [file pbio.3002941.s009.tif]

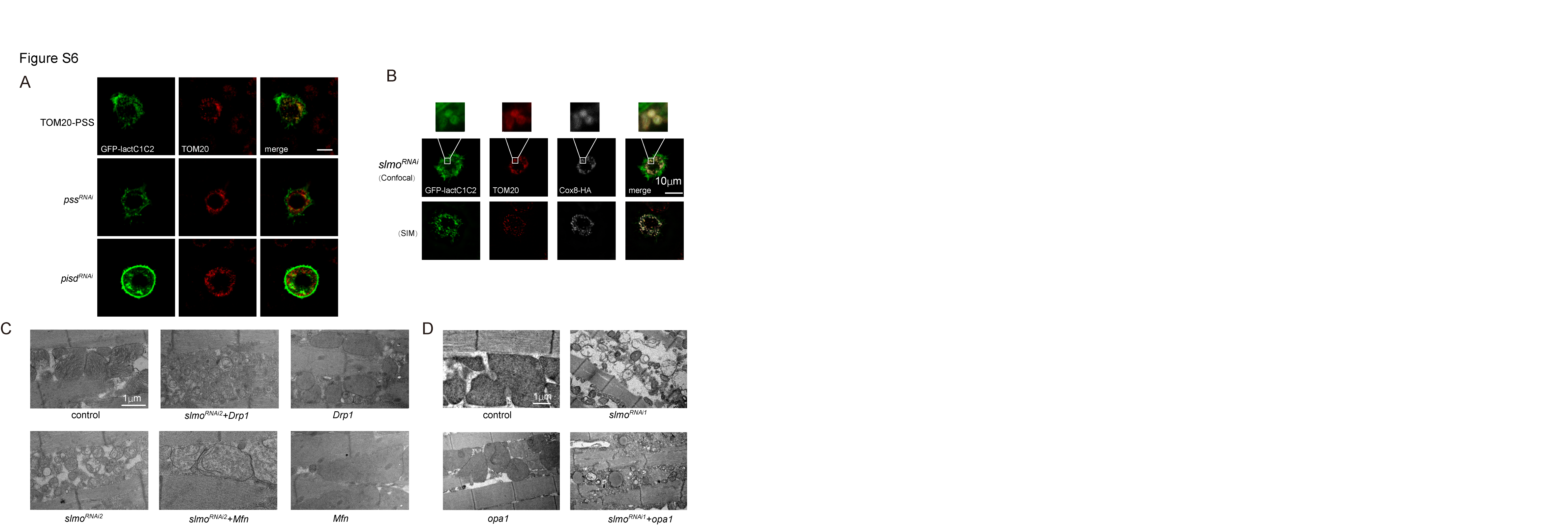

Supplement: S6 Fig — (A) Images of cells transfected with GFP-LactC1C2 (green) with TOM20-PSS-RFP (red) or dsRNA of pss (pssRNAi) or pisd (pisdRNAi). Scale bar, 10 μm. (B) Confocal (up) and Super-resolution SIM microscopy (down) show the localization of LactC1C2, TOM20, and Cox8. S2 cells were transfected with dsRNA of slmoRNAi, pIB-GFP-LactC1C2, and pIB-Cox8-HA, stained by antibody against TOM20 and HA tag. Scale bar, 10 μm. (C) TEM sections of indirect flight muscles of control (MHC-gal4/UAS-GFPRNAi), slmoRNAi2+Drp1 (MHC-gal4/+;UAS-Drp1/UAS-slmoRNAi2), Drp1 (MHC-gal4/UAS-drp1), slmoRNAi2 (MHC-gal4/UAS-slmoRNAi2), and slmoRNAi2+Mfn (MHC-gal4/+;UAS-slmoRNAi2/UAS-marf) and Mfn (MHC-gal4;UAS-marf) flies. Scale bar, 2 μm. All flies were raised for 5 days under 12 h-light/12 h-dark cycles. (D) Muscle section of control (MHC-gal4/UAS-GFPRNAi), slmoRNAi1 (MHC-gal4/+;UAS-slmoRNAi1/+), opa1 (MHC-gal4/+;UAS-opa1/+), and slmoRNAi1+opa1 (MHC-gal4/+;UAS-slmoRNAi2/UAS-opa1) flies. Scale bar, 1 μm. (TIF) [file pbio.3002941.s010.tif]

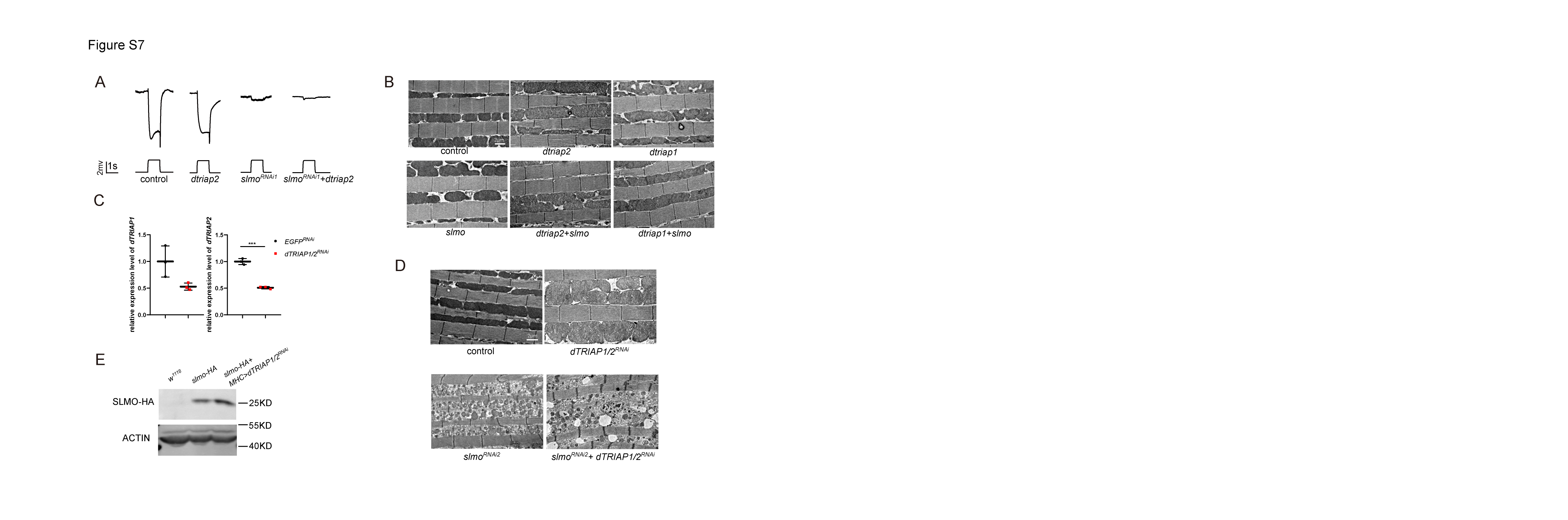

Supplement: S7 Fig — (A) Overexpression of dtriap2 did not affect slmoRNAi. ERG recordings from 5-day-old control (GMR-gal4/UAS-EGFPRNAi), dtriap2 (GMR-gal4/UAS-dtriap2), slmoRNAi1 (GMR-gal4/UAS-slmoRNAi1), and slmoRNAi1+dtriap2 (GMR-gal4/+;UAS-slmoRNAi1/UAS-dtriap2) flies. (B) Co-expression of dTRIAP1/2 and SLMO did not affect mitochondria size. TEM sections from control (MHC-gal4/UAS-RFP), dtriap2 (MHC-gal4/UAS-dtriap2), dtriap2+slmo (MHC-gal4/+;UAS-slmo/UAS-dtriap2), slmo (MHC-gal4/UAS-slmo), dtriap1 (MHC-gal4/UAS-dtriap1), and dtriap1+slmo (MHC-gal4/+;UAS-slmo/UAS-dtriap1) flies. (C) The RNAi efficiency was determined using quantitative Real-Time PCR (qPCR). Total RNA was extracted from the retina dissected from EGFPRNAi (GMR-gal4/+;EGFPRNAi/+) or dtriap1/2RNAi (GMR-gal4/+;UAS-dtriap1/2RNAi) flies. The relative expression of target genes was normalized to RP49, which served as an internal control. Data are presented as mean ± SD, *p < 0.05, ***p < 0.005 (Student’s unpaired t test). n = 3. (D) dTRIAP1/2RNAi failed to reduce the expression of SLMO-HA. SLMO-HA protein levels are indicated using western blotting of 2 thoraxes from 5-day-old w1118, slmo-HA(slmo-HA/+), or slmo-HA+MHC>dTRIAP1/2RNAi (MHC-gal4/slmo-HA;UAS-dTRIAP1/2RNAi) flies. (E) Muscle sections from control (MHC-gal4/UAS-GFPRNAi), dtriap1/2RNAi (MHC-gal4/+;UAS-dtriap1/2RNAi/+), slmoRNAi2 (MHC-gal4/UAS-slmoRNAi2), and slmoRNAi2+dtriap1/2RNAi (MHC-gal4/UAS-slmoRNAi2;UAS-dtriap1/2RNAi/+) flies. The data underlying the graphs shown in the figure can be found in S2 Table. (TIF) [file pbio.3002941.s011.tif]

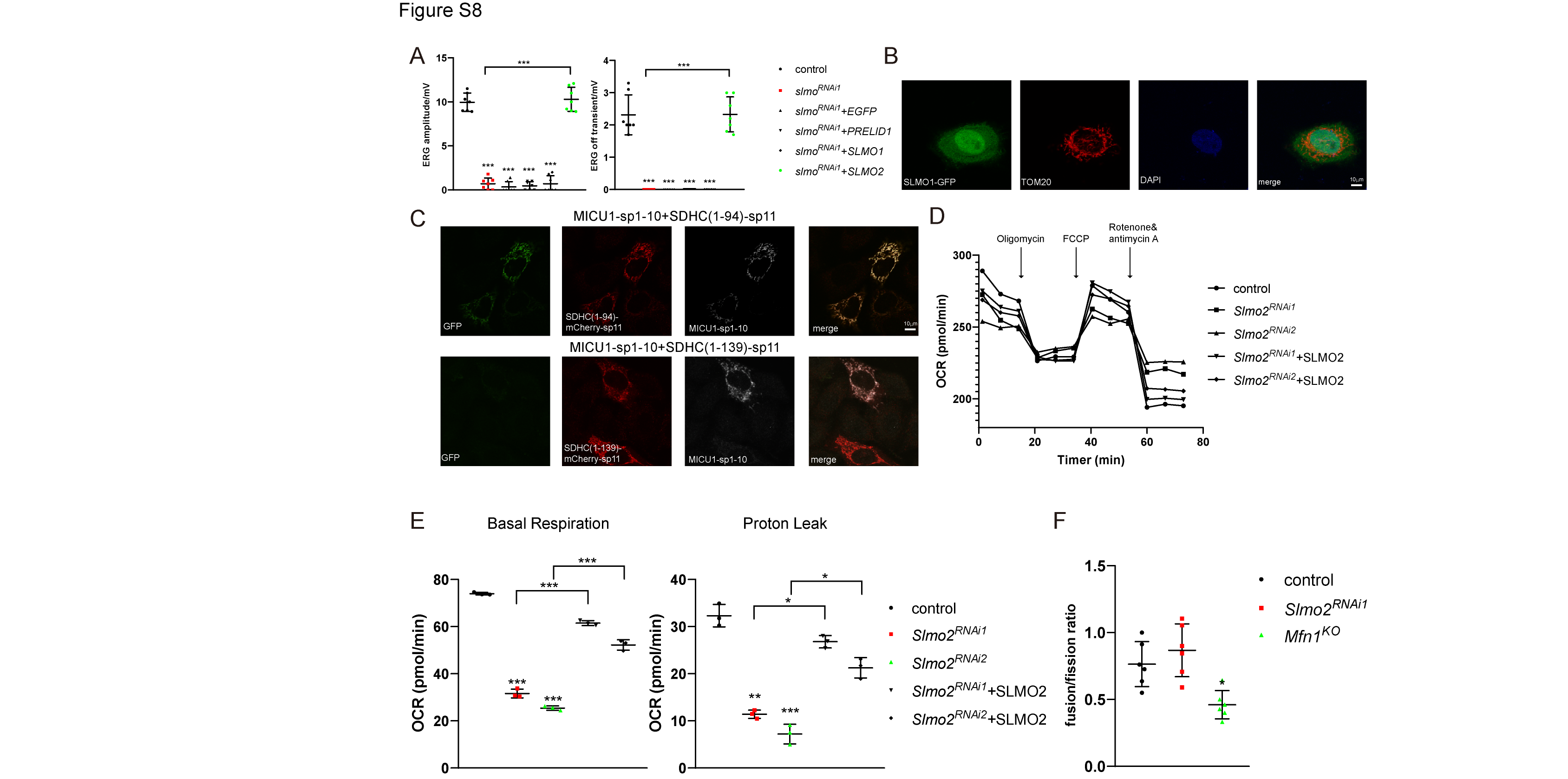

Supplement: S8 Fig — (A) Quantification of the amplitude of ERG responses and off transients from 5-day-old control (GMR-Gal4/UAS-EGFPRNAi), slmoRNAi1 (GMR-Gal4/UAS-slmoRNAi1), slmoRNAi1+EGFP (GMR-Gal4/+;UAS-slmoRNAi1/UAS-EGFP), slmoRNAi+PRELID1 (GMR-Gal4/+;UAS-slmoRNAi1/UAS-PRELID1), slmoRNAi1+SLMO1 (GMR-Gal4/+;UAS-slmoRNAi1/UAS-SLMO1), and slmoRNAi1+SLMO2 (GMR-Gal4/+;UAS-slmoRNAi1/UAS-SLMO2) flies. At least 6 flies of each genotype were analyzed. (B) SLMO1 does not localize to mitochondria. Hela cells were transfected with SLMO1-GFP and stained with TOM20 (red) antibodies. Scale bar, 10 μm. (C) Verification of the split-GFP system in Hela cell. Hela cells co-expressing sp1-10-tagged IMS protein MICU1 (MICU1-sp1-10) with SDHC(1–94)-mCherry-sp11 or SDHC(1–139)-mCherry-sp11 were directly imaged for GFP (green) and mCherry fluorescence (red). Scale bar, 10 μm. (D, E) Oxygen consumption rates (OCR) in Hela cells transfected with Slmo2RNAi1, Slmo2RNAi2, Slmo2RNAi1+SLMO2, and Slmo2RNAi2+SLMO2. Representative kinetics graph indicating real-time OCR at baseline and after addition of oligomycin, carbonyl cyanide p-trifluoromethoxy-phenylhydrazone (FCCP), and rotenone-antimycin (R/A). Basal respiration and proton leak of the indicated genotypes were calculated by normalization of OCR levels to ATP levels. n = 3 wells/group. (F) Quantification of mitochondrial fusion and fission rates in Hela cells. Cells expressing Cox8-GFP transfected with Slmo2RNAi1 and Mfn1 knock-out cells were used as controls. The data underlying the graphs shown in the figure can be found in S2 Table. (TIF) [file pbio.3002941.s012.tif]
