# Supplementary material for: SLMO transfers phosphatidylserine between the outer and inner mitochondrial membrane in Drosophila
Source: PLoS Biol. 2024 Dec 16;22(12):e3002941. doi: 10.1371/journal.pbio.3002941 (PMC11649117; doi:10.1371/journal.pbio.3002941)
Supplement: Attachment — Submitted filename: response to reviewers Zhao et al 20240919.docx [file pbio.3002941.s013.docx]

Rev. 1:

> Zhao et al. studied phospholipid trafficking between the ER and mitochondria using drosophila melanogaster. They focused on mitochondrial PS transport, and using a forward genetic screen identified Slowmo (SLMO) as a PS transporter important for mitochondrial biogenesis. A major criticism with this narrative, is that mammalian isoforms of SLMO (Slmo1 and Slmo2) have already been previously identified as mitochondrial phospholipid transporter in mammals, yet the manuscript barely recognizes this. It is not to say that the paper lacks in novelty: in particular, Figure 5 and 6 shows data on SLMO binding to PS, and the potential non-essential role of TRIAP in drosophila. In general, experiments are well designed, executed, and data presented well, but the whole tone of the manuscript needs to be changed. Authors need to better argue how their findings in drosophila complement what is already known about Slmo2 (Prelid3b) in mammals.

>

> Major comments:

> * The authors overstate the novelty of the study findings as they relate to the existing knowledge of PS mitochondrial import. It is recommended that the authors make substantial revision on the narrative to acknowledge work done by Thomas Langer's group that shows Prelid3b (Slmo2) in mitochondrial PS import.

> We appreciate your suggestion regarding the novelty and significance of this work. In the background section, we now summarize the finding by Thomas Langer’s group that SLMO2/UPS2 is a specific PS transporter in both humans and yeast (lines 50-56). Their comprehensive studies in yeast and mammalian cells have significantly advanced our understanding of the roles of SLMO2/UPS2 and TRIAP1/Mdm35 through biochemistry and structural biology. They demonstrated that the MSF1/PRELI family of proteins are crucial for transferring phospholipids between the inner and outer membranes. This finding was instrumental in guiding our efforts to identify the target protein from our screen. In the revised manuscript, we modified this part and highlighted the identification of SLMO2/Ups2 as PS transfer proteins in both yeast and mammalian cells (lines 56-66).

In response to this suggestion, we also added details about the biochemical functions of SLMO2/Ups2 (lines 148-155) in the result section – right after the identification of slmo in the genetic screen for modifiers of pect. Additionally, we compared Langer’s structural analysis with our Molecular Dynamics simulation. The Asn150 and Thr93 residues that we identified are both conserved in human SLMO2 and yeast UPS2 (new Fig. S5D). Importantly both Asn152 and Ser95 were indicated as key phospholipid binding sites in Thomas Langer's study. We also modified the result section (lines 269-273) and the discussion section (lines 429-434) to discuss the conserved PS transfer activity of SLMO.

In summary, research from Thomas Langer's group has been a valuable source of inspiration and reference for our work. Building on their findings, we have further validated the function of Drosophila SLMO via cell biology and genetics. Moreover, based on these biochemical studies, we generated various slmo mutations to study the fundamental role of PS transfer in mitochondrial morphology and function.

> * Similarly, while cited, Jean Vance's group have substantial data on the role of PSS/PISD to show this pathway is essential for mitochondria. This is largely understated in the manuscript.

We thank the reviewer for raising the concern about discussing the PSS/PSD function studied by Vance’s group. We previously described their work too generally. To improve our manuscript, we added details about the study Jean Vance's group performed regarding the function of PSS/PSD in regulating mitochondrial morphology, highlighting the importance of the PE environment for maintaining mitochondrial structure (Lines 60-66). In the result sections, after we surveyed the different phospholipid pathways that contribute to mitochondrial morphology and function, we described the work of Jean Vance's group on PSD-deficient cells, which is consistent with our results and supports our finding that the transfer of PS to mitochondria is critical for shaping mitochondrial morphology and maintaining mitochondrial function.

Furthermore, we discussed previous studies by the Vance group and others on PISD and PSS in the discussion section (lines 390-405), Briefly, these studies support our conclusions that the generation of PE in mitochondria from PS derived from the ER is fundamental for maintaining mitochondrial size and structure. Our findings explain the severe defects observed in their studies.

> * The main deficient in data for this manuscript are the mitochondrial lipidomic data shown in Figure 1D, but not for any of the other subsequent figures. These quantifications need to be done on every subsequent reiterations of knockdown and re-expression experiments. Without this, authors cannot attribute changes in function or mitochondrial morphology that happens with changes in PSS/SLMO/PISD or TRIAP axes.

> We appreciate the reviewer's attention to the lipidomic data, and we agree that we previously did not pay much attention to this evidence. First, as suggested, we examined the effects on mitochondrial lipids when knocking down *pect*, *pcyt1*, *bbc*, or *pis* (lines 100-107 and new Fig. 1E). We found that knocking down *pect* did not influence mitochondrial PS or PE levels, suggesting that PE from the ER may not be transported to mitochondria under non-stress conditions. However, knocking down *pcyt1* led to a decrease in mitochondrial PC without affecting mitochondrial size, indicating that PC is not the specific lipid determining mitochondrial size. The *bbc* RNAi also did not affect the proportion of each lipid, indicating that it might reduce the generation PC, PE. and PS on the ER. Knocking down *pis* resulted in a loss of PI and a slight decrease in PS, but did not affect PE. These results are described in lines 100-107, and new figures (1E and S1B). In these RNAi experiments, since no phenotypes were observed regarding mitochondrial structure and function, we did not perform rescue experiments.

Regarding Fig. 3A, overexpression of SLMO rescued decreases in mitochondrial size induced by *pss^RNAi^*. We also assessed phospholipid levels (lines 181-185 and Fig. S3A) and found that the loss of PS and PE was rescued by SLMO overexpression or PSS expression, whereas SLMO or PSS overexpression alone had no significant effect on PS/PE levels. Expressing PSS alone increased PS levels but not PE levels, likely due to the saturation of PISD and SLMO. Re-expressing PSS successfully restored PS/PE levels in *pss* RNAi lines.

Finally, we confirmed the epistatic relationship between *slmo^RNAi^* and *psd* via lipidomic experiments, finding that *slmo^RNAi^* caused a significant reduction in PE, which could be fully restored by *trp-pisd* overexpression. These results confirmed our genetic interaction data based on phenotype analysis (Fig S3C and line193-196).

> * Figure 3: how is PISD overexpression rescuing defects induced by Slmo knockdown? If Pisd is downstream of Slmo, PISD overexpression might presumably not rescue any of these phenotype (as there is no mitochondrial PS available as a substrate for PISD). This is one of the reasons why lipidomic data are needed.

Thanks for the concern about the relationship between PISD and SLMO. The reviewer’s speculation is correct. We showed that homozygous slmo null mutation is cell lethal, and that overexpression of *psd* failed to rescue the organismal lethality shown in Fig S2C and the cell lethality demonstrated in Figure S2D. We then used *slmo^RNAi^* as a hypermorph of *slmo* to perform the epistasis test in Fig 3. To confirm the genetic interaction between SLMO and PSD, we performed lipidomic analyses as the reviewer suggested, showing that mitochondria from *slmo^RNAi^* photoreceptor cells exhibited significantly less PE, which could be fully restored by *trp-pisd* overexpression (Fig S3C and line193-196).

> * Figure 5G and S4H: Please also show the colocalization of PS with IMM marker, not just OMM (TOM20). I am not really buying the argument that SLMO deletion promotes accumulation of PSS on OMM according to these images alone.

> We appreciate the suggestion regarding the PS uptake assay in S2 cells. In our view, *slmo* RNAi reduces the transfer of PS to the IMM, leading to PS accumulation on the OMM. The PS reporter, lactC1C2-GFP, which localizes to the cytosol, can only bind with PS on the outer leaflet of OMM but was unable to detect PS within mitochondria (inner leaflet of OMM and IMM). We added the explanation of the lactC1C2-GFP reporter in the manuscript (lines 292-296).

To convince the audience that the localization of PS reporter is on the OMM of the mitochondria, as the reviewer suggested, we imaged lactC1C2 in *slmo* RNAi cells using the IMM marker Cox8, employing both confocal and SIM microscope (Fig. S6A-B, lines 302-306). We observed that Cox8 forms a solid round structure within the lactC1C2-labeled circle, while TOM20 completely merged with lactC1C2. The data confirmed our conclusion that PS accumulated on the OMM upon disruption of *slmo*. However, we currently lack a suitable method to detect PS on the IMM, so we cannot conclude that PS levels changed in the IMM.

> Minor comments:

> * Line 228 please change S5B to S4B

> * Line1213 No schematic is found as indicated in the figure caption

> Thank you for pointing out these mistakes. We have rearranged the order of the figures and carefully reviewed and corrected the legends and captions for each figure.

>

>

>

>

> Rev. 2:

> Mitochondria are essential organelles that have two membranes that differ in their protein and lipid compositions. The mitochondrial inner membrane contains enzymes that result in the production of cardiolipin and phosphatidylethanolamine (PE). Both lipid biosynthetic pathways are fed by precursor phospholipids that originate mainly in the endoplasmic reticulum. In addition, the ER is the source for the other major phospholipid classes that populate mitochondrial membranes but are not made in this organelle. Mechanisms by which mitochondria obtain phospholipids from the ER and move specific phospholipids between the outer and inner membranes have begun to emerge over the past ~15 years. In the present study, Zhao et al provide compelling genetic evidence that the proper synthesis of ER-generated phosphatidylserine (PS) by PSS and its subsequent conversion to PE by PISD in the inner membrane is important for mitochondrial structure and function in Drosophila. To identify

> factors with roles in connecting PSS and PISD activities, a genome wide RNAi screen was performed which identified SLMO, a fly ortholog of the Ups/Preli family which has well documented roles in PS and phosphatidic acid transport from the OM to the IM. Through epistasis analyses, SLMO was placed downstream of PSS and upstream of PISD, and consistent with this placement, shown to reside in the intermembrane space. In yeast and mammals, Ups/Preli proteins heterodimerize with Mdm35/TRIAP1 and this interaction is both important for their lipid transport functions and their stability. In contrast to this expectation, fly SLMO can transport PS between liposomes alone in vitro and genomically HA tagged SLMO steady state amounts and localization to mitochondria are unaffected by the absence of both predicted fly TRIAP proteins. Surprisingly, while both dTRIAP proteins localize to mitochondria, they are associated with the cytosol-facing surface of the OM. Expression of human SLMO2,

> but not PRELID1 or SLMO1, rescued slmoRNAi1 fly phenotypes, suggesting that SLMO2 can also transport PS from the OM to the IM. Hela cells depleted of SLMO2 had smaller mitochondria with aberrant cristae, similar to what was observed in slmo-knockdown flies. Overall, this is an elegant and well controlled study that provides compelling evidence implicating SLMO in the movement of PS into the IM across the intermembrane space. The main twist presented relates to the apparent ability of SLMO to do this solo, an observation with regulatory implications.

>

> Main points:

> 1. Additional controls are needed to support the conclusion that SLMO occupies the inner boundary space. Do import components also give the same staining pattern as SLMO-HA? Figure 4D would benefit from better labelling (e.g which panels represent TOM20 staining).

> We appreciate the suggestion to include additional controls for import into the IMS and labeling. We selected MICU1, a protein known to localize to the mitochondrial IMS and to be involved in Ca^2+^ regulation. The MICU1 pattern appeared as a hollow circle, similar to SLMO, indicating that both proteins are confined to the IMS and do not enter the cristae (Fig. 4D, lines 243-249). In contrast, SDHC, a component of the succinate dehydrogenase complex that is active within the mitochondrial cristae (CM), displayed a solid round pattern, filling the entire cristae structure (Fig. 4E, lines 249-252).

> 2. Lines 219-221: "Using super-resolution microscopy, we found a circular pattern of SLMO-HA that co-localized with TOM20. By contrast, the IMM protein SDHC, which is enriched in the CM region, was surrounded by TOM20 (Fig 4D)." It is unclear whether this data is shown in Figure 4D given that TOM20 panels aren't clearly labeled. If this data is present, it does not support the conclusion that SDHC staining is surrounded by TOM20.

We did not initially stain them together, as our goal was to demonstrate their distinct distributions through different patterns. As the reviewer pointed out, we realize that we may not have clearly explained this. To address this, we have now included co-staining data for ubi-SDHC-mCherry and TOM20 in the new Fig. 4E. In this figure, SDHC-mCherry is surrounded by TOM20, clearly illustrating their spatial relationship (Fig. 4E, lines 247-252).

> 3. Are the 2 SLMO mutants deficient in PS binding as predicted?

We appreciate the reviewer’s suggestion to examine the ability of SLMO mutants to bind PS. Our goal was to generate a site-specific mutation that disrupts the PS transfer ability of SLMO. Therefore, we performed the PS transfer assay. As the reviewer suggested, we assessed the binding ability of mutants using a Liposome Flotation assay. Liposomes containing PS bound nearly 80% of MBP-SLMO proteins, whereas both the T93A and N150A mutants showed reduced levels of proteins in the liposome fraction, which is similar to the MBP control. This suggests that both T93A and N150A mutations largely reduce the ability of SLMO to bind PS. We incorporated these data into new Fig. S5E and described these experiments in the result section (lines 269-273).

> 4. Given that the Ups/Preli family from yeast and mammals are short-lived due to their continuous degradation by Yme1, this would suggest that this pathway is regulated in a distinct manner in flies. Given this difference, it might be worth adding this possibility to the discussion section.

We appreciate the reviewer’s suggestion to discuss Yme1. In the revised manuscript we added a few sentences in the Discussion section to discuss the relationship between SLMO, Yme1, and dTRIAP1 (lines 446-455).

> 5. Is the PE percentage in mitochondria really less than 1% of total phospholipids? And the amount of PC is less than PE? The amount of each seems very low and inconsistent with the contention that PE and PC are major building blocks of membranes. If PE and PC make up less than 2% of mitochondrial lipids, what are the main components of its membranes? Some explanation for these values would be helpful.

We thank the reviewer for raising the issue about the percentage of various phospholipids. In our Figures, a value of 0.4 on the ordinate corresponds to 40%, which may mislead the reader. For example, in Figure 1, PE makes up about 50%-60% of total phospholipids, whereas PC accounts for about 30%-40%. In the Figure, however, PE counted for 0.5-0.6 and PC counted for 0.3-0.4. To avoid this misunderstanding, we changed the percentage to a hundred-mark system. In Figure 1, the Y axis indicating % phospholipid levels is labeled with “%” (Fig 1D, 1E, 5F, 6D, S3A and S3C).

In our study of fly muscle, we observed that PE is indeed more abundant than PC, likely due to the high content of CM within muscle mitochondria. This would result in the outer membrane phospholipids representing a relatively smaller proportion. The IMM is typically rich in PE, with less PC, which contrasts with reports from other cell types where PC is more prevalent than PE.

> Minor points:

> 1. The GFP signal in Fig. 7D is pretty weak compared to the other split-GFP-based data. Perhaps a more convincing image could be provided.

> Thanks for pointing out the weak signal in Fig D. We repeated the experiments and added new pictures.

> 2. Line 228: (Fig. S5B) should be changed to (Fig. S4B).

> Thanks for pointing out this mistake. We have rearranged the order of the figures and carefully reviewed and corrected these kinds of mistakes

> 3. Line 229: This assay seems like a liposome floatation assay, not a liposome sediment assay. Also, I could not find the method for this assay (Fig. s4c).

It is correct that we used a liposome flotation assay. We changed the name of the assay to “liposome flotation assay” and now include a detailed method.

> 4. Abstract: Would add "putative" to sentence: "SLMO is required for shaping mitochondrial morphology, but its putative conserved binding partner, dTRIAP, is not." This better reflects the authors'discovery that both dTriap proteins are on the OM and that SLMO protein levels are insensitive to their combined absence.

Thanks for this advice regarding the abstract; we have added “putative” to the sentence.

> 5. Lines 45-47: This statement is incorrect as mitochondria are the sole organelle that makes cardiolipin. Suggest changing "The only" to "An exception".

>

It is correct that CL is also synthesized in mitochondria, which we previously overlooked. In the revised manuscript we have corrected this sentence to: “An exception is phosphatidylethanolamine (PE), as most mitochondrial PE is derived from imported PS.”

> 6. Lines 64-66: This statement is also incorrect as several papers have assessed mitochondrial morphology and function in the context of perturbed phospholipid movement into and across the mitochondrial intermembrane space, including several studies that the authors' have cited (PMID: 23042293; PMID: 27241913; PMID: 29301859; PMID: 23931759; PMID: 30650346).

We thank the reviewer for pointing out this confusing statement. We have rewritten this part of the introduction and deleted this sentence (lines 50-66).

> 7. For the non-fly expert, it would be helpful if the EM images were labeled more thoroughly (e.g. the rhabdomeres and photoreceptor cells; mitochondria and sarcomeres?).

>We added labels to several Figures to point out rhabdomeres (r) in photoreceptor cells (Fig 2C), as well as the mitochondria (M), and myofibrils (F) in muscle cells (Fig 1B, 2E, 3A, 5D, and 6B). We also added a cartoon illustrating the structure of a single ommatidium in Fig 2C.

> 8. What side of the IM is the GFP tag attached to SDHC.

According to previous studies, SDHC is a transmembrane protein with three transmembrane domains and its N-terminal in the IMS. A SDHC fragment (comprising amino acids 1-94) with a C-terminal GFP tag localized to the IMM facing the IMS, whereas full-length SDHC(1-139) with a C-terminal GFP tag extended into the matrix. We tested these two truncated forms using the split-GFP system. In our *in vivo* digitonin assay, we used SDHC(1-94) with a C-terminal GFP tag as the control, in which GFP is in the IMB region. In the revised manuscript we now clarify this (line 251, the CM protein SDHC (SDHC(1-94)-GFP or mCherry)). We also clarify this point in the legend of Fig 4D and 4E.

>

>

> Rev. 3:

> Zhao et al. utilized a forward genetic screen in Drosophila to explore how PS-PE production in mitochondria impacts mitochondrial function and structure. They identified the protein Slowmo (SLMO) from a sensitized background. Through microscopy, ERG, TEM, and lipidomic analysis, SLMO was found to be crucial for PSS/PISD in maintaining mitochondrial structure and function. They demonstrated that SLMO specifically transfers PS from the outer mitochondrial membrane to the inner mitochondrial membrane via an in vitro lipid transfer/binding assay. This transport is vital for mitochondrial morphology, independent of mitochondrial dynamics. The authors further showed that the mammalian homologue SLMO2 is functionally conserved. The manuscript is well-written, with clearly presented data and well-controlled experiments. Combined with the smooth logical flow, this makes the manuscript an enjoyable read. This will be of interest to those working in related fields.

>

> Some minor points:

> - Why is PC increased in 1D? Can the authors explain the potential cause?

We thank the reviewer for their question regarding the *pisd/pss/slmo* RNAi lines. PC and PE together make up the majority of mitochondrial phospholipids. A reduction in the relative levels of PE naturally leads to an increased proportion of PC. However, this does not imply that the absolute amount of PC has increased. Rather, a decrease in PE can result in smaller individual mitochondria and disrupted cristae structure, even if the absolute amount of PC remains unchanged. Similarly, knocking down *pcyt1* reduced PC levels, which of course raised the proportion of PE in mitochondria.

In the revised manuscript, we provided the lipidomic data upon disruption of the PC pathway by *pcyt1^RNAi^*, and disruption of both PC and PE pathway by *bbc^RNAi^.* As results, knocking down *pcyt1* decreased mitochondrial PC levels, and thus reduced PE levels. In contrast, knocking down *bbc* did not change phospholipid rations, because BBC is required for the synthesis of most phospholipids on the ER (Fig 1E, lines 100-107). These data give clear examples of the relationship of the mitochondrial PC/PE.

> - The paragraph describing the epistatic relationship between SLMO and PSS/PISD (line 161 onward) seems weaker compared to other data. For example, the fact that slmoRNAi+PISD rescues the slmoRNAi phenotype does not eliminate the possibility that they function in parallel pathways rather than in an upstream/downstream relationship. I wonder whether the authors could show SLMO(OE)+PISD RNAi to further support their conclusion (the same applies to SLMO and PSS). Alternatively, the authors could modify the manuscript to acknowledge this possibility.

We thank the reviewer for these suggestions. It is correct that in photoreceptor cells, the alternative explanation for the result that ERG defects caused by *slmo^RNAi^* can be rescued by *trp-pisd*, is that SLMO and PISD function in parallel pathways. To rule this out, as the reviewer suggested, we examined whether overexpression of PISD could affect *pisd ^RNAi^* phenotypes. Since knocking down *pisd* in the retina did not produce a phenotype, we performed the experiment in flight muscles. Overexpression of SLMO did not affect the reduced mitochondrial size in muscles expressing *pisd^RNA^*. These data provided more genetic evident that SLMO acts upstream of PISD in regulating mitochondrial morphology. We added these data to a new figure (Figure S3B) and updated the text (lines 193-197).

> - The authors claimed that SLMO-GFP does not colocalize with TOM20 in mature sperm after elongation, due to the lack of a proper mitochondrial marker in the mature sperm tail. This description is somewhat tautological. Perhaps rephrase this explanation? (If the authors choose to present this, please add an arrow in Figure 4A to indicate the mature sperm.)

We apologize for our previous lack of clarity. Mitochondria undergo five key steps during sperm formation: aggregation, fusion, membrane wrapping, unfurling, and elongation. After meiosis, mitochondria aggregate and fuse, forming an onion-shaped structure called the nebenkern. This process relies on the proper functioning of mitochondrial fusion and fission machinery.

Our staining results show that both TOM20 and SLMO signals co-localize before the onion stage and are enriched in the onion stage (as indicated by white arrows). Following this, mitochondrial elongation occurs along cytoplasmic microtubules, causing mitochondria to become filamentous. We found that the TOM20 signal was almost undetectable in filamentous mitochondria (due to the reorganization of mitochondria) whereas the SLMO signal persisted. Because Tom20 is not detectable in mature sperm, the statement that “SLMO-GFP does not colocalize with TOM20” is not entirely accurate. Therefore, we deleted this statement and wrote a few sentences to explain this observation. Please check the revised text for details (lines 208-217). We also add asterisks to mark elongated mitochondria in mature spermatozoa (Fig 4A).

> - The data supporting the localization of SLMO in IBM seem to be missing (line 219 and Fig 4D). Please elaborate on the logic or data that support the idea (especially focus on how they can distinguish cristae vs IBM).

> We thank the reviewer for pointing out these issues regarding IBS vs. CM. Due to resolution limitations, we cannot distinguish between OMM and IBS proteins using light microscopy. As a result, SLMO and TOM20, which both localize to the mitochondrial outer membrane, appear as hollow circles. In contrast, CM proteins can be distinguished from IBS-localized proteins. For example, SDHC, which localizes to mitochondrial cristae, forms solid circles when viewed with confocal or SIM microscopy. To provide the logic and data to support this, we cited a paper on MICU1, which localizes to the IBS to facilitate rapid uptake of Ca^2+^ by mitochondria (PMID: 31427612). Knowing that MICU1 localizes to the IBS, we examined MICU1 protein localization in muscle cells using SIM microscopy. We found that the circular pattern of MICU1-sp1-10 co-localized with TOM20, whereas the CM protein SDHC formed a solid circle surrounded by TOM20. This provided the control data that allowed us to distinguish between the IMS and CM. We now include co-staining data for MICU1-sp1-10 and SDHC-mCherry with TOM20 in a new figure (Fig. 4D and 4E) to provide support information (lines 243-249).

> - Are the SLMO T93 and N150 residues conserved? Can the authors include some alignments? Are there known diseases associated with mutations in these residues?

Both Asn150 and Thr93 (Ser in SLMO2 and Ups2) residues are conserved in human SLMO2 and yeast Ups2. We have included these alignments in Fig. S5D and detailed them in lines 269-273. Additionally, we compared the structural analysis from Thomas Langer's group with our Molecular Dynamics simulation. Importantly both Asn152 and Ser95 were indicated as key phospholipid binding sites in Thomas Langer's study. We also modified the discussion section (line 428-433) to discuss the conserved PS transfer activity of SLMO. We have not yet found reports of a disease linked to point mutations in SLMO2, which may be because *slmo* null mutations result in cell lethality.

> - The PS binding assay uses LactC1C2 as a reporter. Can the authors provide more information about which part of PS it binds/recognizes (e.g., head group)? If I understood correctly, the LactC1C2 was expressed without a mitochondrial targeting sequence, limiting the reporter to detect only the cytoplasmic side of the lipid bilayer of the OMM. The authors should make such information more explicit in writing.

We appreciate the reviewer’s concern regarding the structure of the lactC1C2 domain. Based on computational docking of phosphatidyl-L-serine (PS) with Lact-C2 and previously crystallized protein-phospholipid complexes, the head group of PS is identified as the primary binding site. PS molecules are oriented such that their acyl chains are embedded in the hydrophobic core of the membrane, whereas the polar head groups interact with the polar residues of Lact-C2. In our assay, because LactC1C2 localized to the cytosol, it was only able to bind PS that were oriented towards the cytosol, such as PS on the outer leaflet endomembrane system and the inner leaflet of the plasma membrane. Therefore, we can detect PS on the outer leaflet of the OMM and the inner leaflet of the plasma membrane using this LactC1C2 reporter. We added a couple sentences to now describe the molecular basis of the LactC1C2 reporter and the rationale for detecting PS in our cell system by expressing the LactC1C2 reporter (lines 292-296).

> - (Line 267) How did the authors conclude that PS trafficking to the plasma membrane is not affected by SLMO-KD? To interpret this alongside Figure S4H, why does PSS KD cause increased PS on the OMM and PISD KD cause increased PS in the plasma membrane? If this conclusion is based on confocal images, please include arrows and/or quantifications.

We thank the reviewer for raising this issue. Quantifying the amount of PS based on fluorescence intensity alone is not accurate, and we can only draw conclusions about the ratio of PS in individual membrane systems. Therefore, we did not perform a quantitative analysis of fluorescence intensity but focused on qualitatively assessing the percentage of LactC1C2 signals on mitochondria, which reflects the relative amount of PS on the OMM. In normal conditions, PS is enriched in the inner leaf of the plasma membrane, and LactC1C2-GFP is indeed enriched at the plasma membrane. Upon loss of SLMO, a large portion of LactC1C2-GFP moved to mitochondria, with the rest remaining in the plasma membrane. Therefore, we concluded that PS trafficking to the plasma membrane was not affected. Since we cannot quantify PS levels, and to avoid overstatement, we took out the sentence “Notably, the distribution of PS in the plasma membrane could still be detected in slmoRNAi, suggesting that SLMO is not involved in PS synthesis and trafficking to the plasma membrane.” We have added the CM protein, Cox8, as an additional control for mitochondria, and rewritten the sentence (Fig. S6B, lines 303-306).
